# Comparative Analysis of Reported Gene Targets and Binding Regions of Glu-CTC tRNA Fragments Across Human Diseases

**DOI:** 10.3390/biom16091313

**Published:** 2026-09-10

**Authors:** Nikita Gulati, Andrey Grigoriev

**Affiliations:** 1 Department of Biology, Rutgers University, Camden, NJ 08102, USA; 2 Center for Computational and Integrative Biology, Rutgers University, Camden, NJ 08102, USA

**Keywords:** tRNA-derived fragments, tRF-Glu-CTC, tRF databases, target recognition, gene regulation

## Abstract

Recurrent detection of overlapping tRNA-derived fragments (tRFs) across diverse disease conditions supports the emerging view that tRFs may act as regulatory molecules rather than random degradation products. While cases of identical tRFs have been described, their comparative analyses are lacking. tRF-Glu-CTC is one such fragment, repeatedly detected in various pathological conditions. We performed a comparative analysis of tRF-Glu-CTC isoforms, their targets and binding regions reported in 18 disease-associated studies. An 18-nucleotide sequence, TCCCTGGTGGTCTAGTGG, was identified in most (14 out of 18) of these studies despite differences in tRF naming, length and disease context. Several reported tRF targets showed consistent binding regions, with reverse complementarity to the tRF sequence. Comparison with databases of tRF targets, tatDB and tRFTar, identified matching target entries and sequence overlaps, often involving common regions rather than full-length matches. Exploratory analysis of target homologs further illustrated that related genes might share candidate target sites. Our findings indicate that tRF-Glu-CTC represents a recurrent candidate regulatory fragment potentially relevant in a broad range of human diseases. Its structural stability, extracellular vesicle association, detection in multiple species and a core sequence shared between related isoforms support further investigation of its biological and translational relevance. Our work illustrates how tRF target databases can be leveraged to advance smaller-scale tRF studies.

## 1. Introduction

tRNA-derived fragments (tRFs) are a distinct class of small noncoding RNAs with potential regulatory functions. Although they were initially considered random degradation products, many studies have shown that tRFs can be generated in a regulated manner. They participate in cellular processes such as post-transcriptional gene regulation, translation control, apoptosis, stress response, immune signaling and disease-associated pathways [1,2,3,4]. Several tRFs are evolutionarily conserved and are associated with Argonaute proteins, suggesting that they may function through mechanisms similar to those of other small regulatory noncoding RNAs [5]. Similar tRF species are detected in multiple organisms and exhibit selective Argonaute association, supporting the idea that their accumulation and function are biologically organized rather than incidental [6].

**Figure 1:**
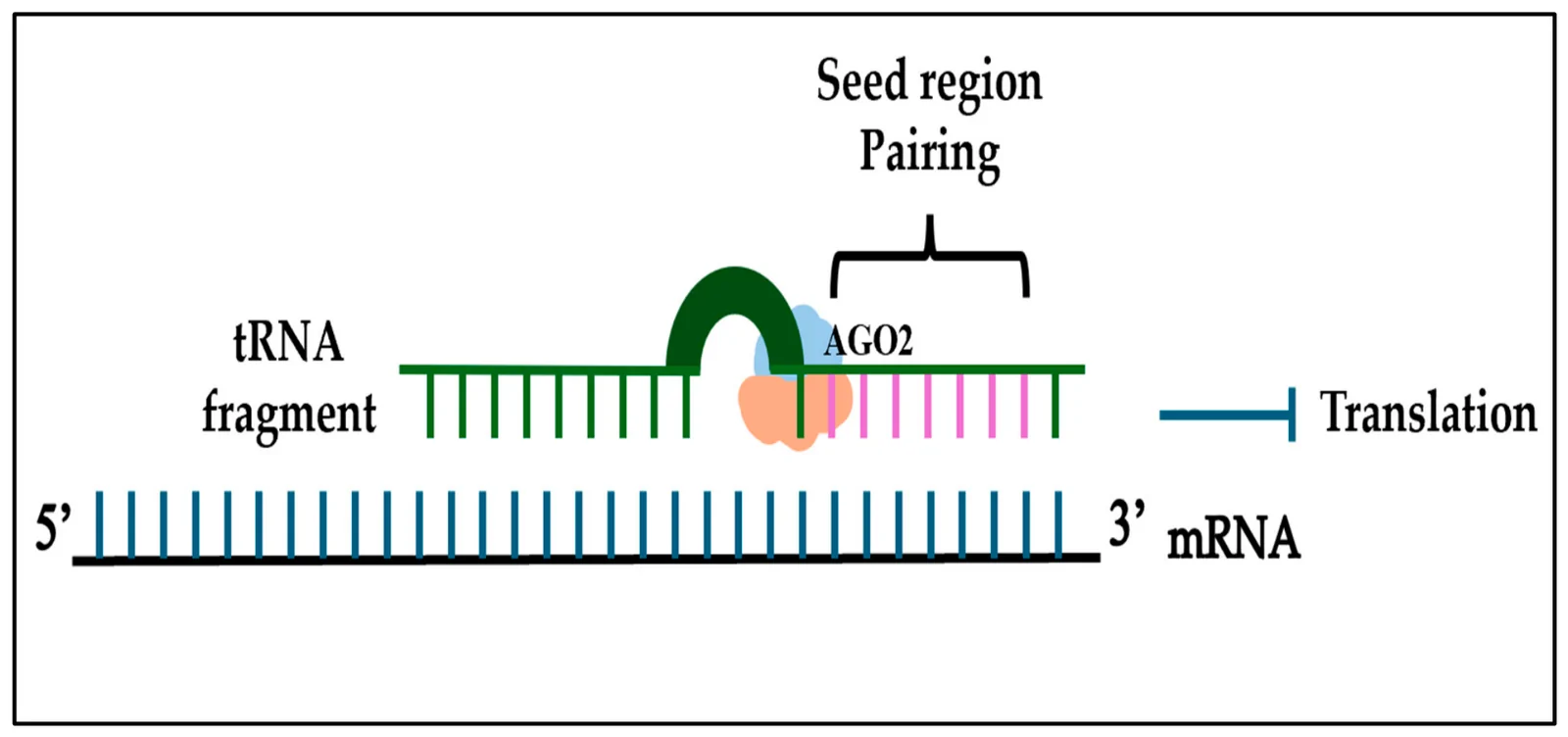
Conceptual model of possible miRNA-like tRF-guided Argonaute-containing silencing complex interaction with a complementary region of mRNA. Four human Argonaute proteins (AGO1–AGO4) are known; each of them may serve as a core component of the RNA-induced silencing complex (RISC). In this example, AGO2 is shown. To illustrate the similarity with the canonical miRNA mode of action, the seed region is indicated at positions 2–8 (purple color) from the 5′ end of the tRF, following the seed pairing principle. The interaction contributes to post-transcriptional regulation of the target transcript, e.g., leading to repression of translation, as shown here. The illustrated miRNA-like seed model represents one possible mechanism and is not universal. tRF regulatory mechanisms may also be noncanonical and context-dependent.

Similar to microRNAs (miRNAs), some tRFs can regulate gene expression through sequence-specific interactions with target mRNAs, among other mechanisms, as we reviewed earlier [7,8]. A short seed-like region within a tRF may guide an Argonaute-containing silencing complex toward complementary target regions (Figure 1).

In addition to sequence-guided targeting, biological persistence of certain tRFs may be influenced by their structural properties. 5′ halves derived from tRNA-Gly can form homodimers, as well as heterodimers with 5′ halves derived from tRNA-Glu, increasing their stability, while disruption of dimerization increases their susceptibility to nuclease degradation [9]. Thus, intermolecular RNA structure can enhance tRF stability and may help explain why selected Glu- and Gly-derived fragments are repeatedly detected in extracellular RNA populations [9].

Due to the limited number of experimentally validated tRF–target interactions, there remains a significant knowledge gap in this field. Most studies focus on one or two tRFs in isolation, often lacking functional assays to confirm predicted targets, which limits mechanistic insight. This prevents broader understanding of whether recurrent tRFs act through a shared molecular mechanism or on context-specific targets [10]. However, large-scale experiments have also been performed on small RNAs and their results are available in databases, such as tatDB [11], developed in our laboratory, and tRFTar [12], which can further support single-tRF studies. tatDB contains our earlier large-scale analysis of experimentally detected tRF-mRNA hybrids and was used in this comparative assessment as independent support of target binding observations.

The CLASH (Crosslinking, Ligation, and Sequencing of Hybrids) protocol was initially designed and performed to capture miRNA–target interactions associated with Argonaute (AGO) proteins [13]. The CLEAR (Covalent Ligation of Endogenous Argonaute-bound RNAs)-CLIP experiments enhanced standard CLIP methods by elucidating the connections between RNA and RNA-binding proteins, such as AGO, while also ligating the RNA to the target [14]. tatDB [11] provides results of integrated analyses [4] of large-scale CLASH experiments [13] for AGO-mediated tRF-mRNA interaction, including target genes, binding motifs and RNA hybrid structures [11]. tRFTar [12] offers complementary predictions based on machine learning models trained on CLASH and CLEAR-CLIP data [14] but the prediction reliability is unknown. These databases are useful for target comparison, but their target reports may vary because of experimental context, transcript isoform coverage, tRF length differences, tissue specificity and database-specific filtering.

The extracellular behavior of tRFs is also increasingly relevant to disease studies. tRFs are actively enriched in extracellular vesicles and delivered to recipient cells in a concentration-dependent manner [15]. Because stability can influence persistence in extracellular compartments and recovery from biofluids, recurrent and structurally resilient tRFs may be more readily detected in biomarker-oriented investigations. Recent disease-focused studies have reported cases where identical or highly overlapping tRF sequences appear in different disease contexts [10]. This raises the important question of whether recurrent tRF sequences reflect shared molecular mechanisms, common disease-associated pathways, or incidental reuse of similar sequence fragments.

Our previous CLASH-based motif analysis of tRF-mRNA hybrids supported a motif-guided model of tRF targeting and classified tRF-mRNA hybrids by sequence motifs, unique target genes, CLASH reads and unique hybrid counts [4]. tRFs derived from tRNA-Glu-CTC showed particularly high target representation in the CLASH-based analysis [16], providing a rationale for examining this fragment in greater detail. Therefore, in the present study, tRF-Glu-CTC was selected as a focused candidate as its identical or highly overlapping sequences were described in a broad range of human diseases. Eighteen studies reporting tRF-Glu-CTC detection were thoroughly screened and compared for reported tRF sequence, disease context, validation method, target gene, binding region and database support. Further, the repeated tRF-Glu-CTC sequences and their targets mentioned were compared with the contents of tatDB and tRFTar databases and we assessed whether reported or predicted target genes share reverse complementary target site features.

## 2. Materials and Methods

### 2.1. Finding Recurrent tRF-Glu-CTC Sequences

A focused literature-based analysis was conducted to identify diseases associated with tRFs derived from glutamic acid tRNAs with the CTC anticodon. The initial PubMed search used the term “tRF-Glu-CTC” (https://pubmed.ncbi.nlm.nih.gov/?term=tRF-Glu-CTC; last accessed 15 August 2026) followed by iterative searches using combinations of terms such as Glu-CTC tRNA fragment, glutamic acid tRNA fragment, tRNA-Glu-CTC-derived fragment, 5′ tRF-Glu-CTC, 3′ tRF-Glu-CTC and related names reported in individual studies. This search was complemented by similar queries in ScienceDirect. In total, it covered studies available up to May 2026, with no predefined starting publication date. Retrieved studies were screened manually for reports of Glu-CTC tRFs or closely overlapping Glu-CTC sequences. Studies were deemed eligible and included when they provided disease-associated information or target-related evidence relevant to the comparative analysis. Studies were excluded if they did not report Glu-CTC-derived tRFs or closely overlapping sequences or lacked disease-associated information or target-related evidence relevant to the comparative analysis. For the included studies, the reported tRF sequence, disease context, target gene, validation approach, and target binding information were extracted. Studies reporting tRF-Glu-CTC or closely related Glu-derived tRFs, or similar sequences, were screened, with particular emphasis on fragments showing identical or highly overlapping sequence regions. Sequence comparisons were used to evaluate whether differently named tRFs reported across disease studies may correspond to closely related or overlapping tRF-Glu-CTC isoforms. Studies were retained even when the naming convention of the fragment differed, provided that the reported sequence overlapped with the recurrent tRF-Glu-CTC sequence region identified during the analysis. Database comparisons were performed using tatDB (version 1.0) and tRFTar (no version on website).

### 2.2. Comparing Small-Scale Results (Literature) with Large-Scale Experiments (Databases)

Each tRF-Glu-CTC sequence was cross-referenced in tatDB and tRFTar. tatDB was used to evaluate large-scale tRF-mRNA interaction evidence from CLASH experiments initially designed to capture miRNA–target interactions associated with AGO proteins [13]. tatDB was queried using tRF sequences and target gene names to retrieve tRF isoforms, target gene associations, hybrid read information, sequence logo patterns and estimated minimum free energy for tRF–target duplex. All this provided independent binding evidence from analyzed CLASH interactions. tRFTar was queried using the tRF sequences and target gene names to identify corresponding predicted and curated tRF–target associations. The T1–T3 evidence tiers were used to classify the strength of target support reported in the original literature studies and were considered separately from database-based comparisons. Partial overlaps between literature-reported sequences and database tRFs were considered, including database tRF isoforms that were truncated or extended relative to the literature-reported sequence. tRF sequence overlaps were defined by exact nucleotide identity within the overlapping region, with mismatches disallowed. Full-length sequence identity was not required because reported tRF isoforms may differ in their 5′ or 3′ boundaries; therefore, truncated and extended isoforms were considered when they shared an exact overlapping sequence. We started with a stringent 15 nt overlap threshold (~3 random occurrences in the human genome). Following comparison of the literature-reported tRF-Glu-CTC sequences, an 18 nt sequence shared across multiple reported fragments was observed as a recurrent region, with one exception of 9 nt as described in Results. This 18 nt region was identified after study selection and sequence comparison and was not used as a prespecified criterion for inclusion of studies or tRF sequences.

### 2.3. Analysis of Recurrent tRF Sequences and Target Binding Site Regions

We examined whether recurrent tRF-Glu-CTC sequences were associated with common target site features in unrelated genes and disease settings. Both the tRF-Glu-CTC sequence and the corresponding reported target binding regions were evaluated for recurrent shared sequences. Binding region information was collected from original disease studies whenever available. When papers provided a target interaction region, those regions were compared with the corresponding target binding sequences retrieved from tatDB and tRFTar. This comparison was used to assess whether the diverse literature-reported targets contained similar complementary sequence features despite differences in disease context or tRF isoform length. Database-derived examples were used as complementary evidence to interpret whether common tRF-Glu-CTC sequence regions were associated with plausible target interactions and to compare these findings with reported target relationships.

### 2.4. Homolog-Based Comparison of Candidate Target Site Similarity

To investigate cases in which the literature-reported target genes and database-supported entries showed incomplete agreement, selected family members or homologous genes were examined further. For example, 3′UTRs of AKT homologs were retrieved from NCBI transcript records and aligned to compare potential target site regions centered on CCAGG. This analysis was used as an exploratory approach to distinguish direct database-supported targets from related genes that may represent homology-based candidate targets. Sequence similarity of a candidate binding region was not considered evidence of functional tRF-mRNA interaction without direct experimental validation. We selected AKT family genes because both AKT1 and AKT2 were reported as targets of tRF-Glu-CTC. RefSeq mRNA records were retrieved for AKT1 (NM_001382430.1), AKT2 (NM_001626.6) and AKT3 (NM_005465.7). The corresponding 3′UTR sequences were obtained from the annotated RefSeq transcript records. The 3′UTRs were searched for candidate complementary sequences corresponding to the reported tRF binding region (ACCAGGGA, CCAGGG and ACCAGG-related sequences, including the shared CCAGG core). For each candidate site, ±50 bp flanking sequences were extracted for multiple sequence alignment using Clustal Omega (https://www.ebi.ac.uk/jdispatcher/msa/clustalo; last accessed 14 July 2026) with default parameters.

## 3. Results

Using the approaches above, we found multiple publications describing potential involvement of tRF-Glu-CTC in various diseases. A total of 18 studies were selected for comparative analysis with the information of reported tRF sequence, target gene information, disease association, validation method and target binding region (Table 1 and Table 2). Detailed descriptions of gene functions corresponding to the reported targets in Table 1 are provided in Table A1 (Appendix A).

### 3.1. Recurrent tRF-Glu-CTC Sequences Share an 18 Nt Core in Disease Studies

Consistent with the previous observations that identical tRF sequences can be found in distinct pathological contexts [10], a recurrent core sequence of 18 nucleotides (nt) TCCCTGGTGGTCTAGTGG was identified in a wide spectrum of diseases, including carcinogenesis, metabolic and neurodegenerative, viral infections and vascular remodeling. Despite variation in naming conventions (Table 1), these fragments contain highly overlapping sequence stretches (Table 2). The level of target evidence varied among studies, ranging from experimental validation to expression-based support and bioinformatic prediction. Most studies included experimentally validated results, while a few relied just on miRNA-based tools for target prediction.

In the 18 studies included in this analysis, the recurrent fragment was reported under multiple nomenclature systems, including names like tRF-GluCTC-0005, tRF-Glu-CTC-014, tRF-Glu-CTC and tDR-001276. We have already discussed the issues arising from the divergent tRF naming systems [7]. However, sequence comparisons showed that these differently named fragments frequently retain the same 18 nt core, indicating that nomenclature differences may obscure recurrence of closely related or identical tRF species in disease contexts.

During manual curation of the included studies, variation was observed in tRF nomenclature, reported fragment length, sequence boundaries and reporting format across the literature and their supplemental material. For example, tRF-Glu-CTC-005 was identified as a Glu-CTC-derived fragment and used for sequencing, RT-qPCR validation and target prediction, although its exact nucleotide sequence was not explicitly reported [31]. Our detection of the same target of tRF-Glu-CTC is thus based on tatDB search by amino acid and codon; interestingly, it results in the same 18 mer sequence. In another case, the reported tDR-001276 sequence [23] showed strong overlap with the recurrent tRF-Glu-CTC sequence identified in our analysis and was therefore retained based on sequence overlap, although its corresponding standardized tRF annotation was found to be Glu-TTC. In some individual papers, differences were observed between sequence lengths or representations provided in text, figures, supplementary tables and experimental constructs, reflecting variation in sequence boundary definitions across studies. Importantly, even in cases with nomenclature or sequence reporting differences, CLASH-based tatDB provided independent support for binding of some of the reported targets by tRFs containing the common recurrent sequence. These reporting differences highlight the importance of manual sequence-level curation when comparing tRF studies across different nomenclature systems and experimental settings.

### 3.2. Reported Targets Relate to Diverse Disease Contexts, Yet Binding Region Information Is Uneven in Studies

The disease spectrum (Table 1) further indicates that this regulatory fragment is not limited to a single tissue type or pathological category. Instead, it appears in vascular, oncologic, neurodegenerative, inflammatory, reproductive, infectious and metabolic settings, prompting us to investigate whether a shared sequence is accompanied by recurrent targeting patterns.

**Figure 2:**
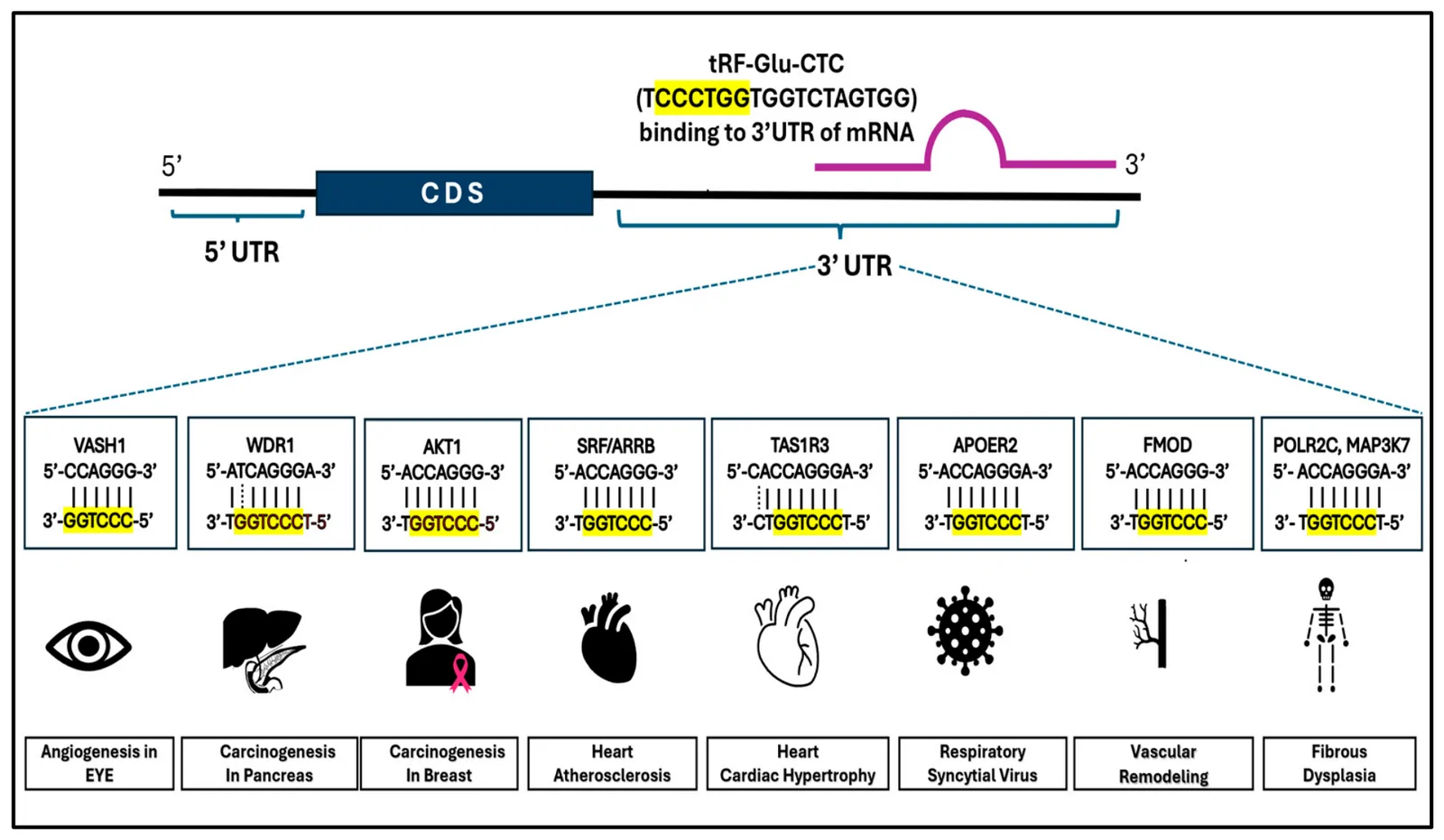
Schematic representation of tRF-Glu-CTC target binding regions in different disease contexts. The (**upper**) panel shows mRNA structure illustrating tRF-Glu-CTC binding to 3′UTR. The recurrent identical sequence within tRF-Glu-CTC is highlighted in yellow. The (**lower**) panel shows reported 3′UTR binding regions for targets whose binding sites were provided in the original studies along with reported disease associations. Corresponding database support was identified for most of these targets; however, APOER2, TAS1R3 and FMOD were not found as direct target matches in tatDB or tRFTar under the stated criteria. In each box, the upper sequence represents mRNA target site in 5′ to 3′ direction, and lower sequence represents complementary tRF sequence in 3′ to 5′ direction. Yellow highlighting indicates the identical sequence. Solid vertical lines indicate complementary base pairing, whereas dashed vertical lines indicate wobble base pairing.

We observed recurrent complementary sequences in several reported interactions (Table 2). Overlapping binding regions (CCAGGG, ACCAGGG, ACCAGGGA) were reported in these targets (Figure 2). Notably, the recurrent seed-like region spans positions 2–8 of the shared tRF-Glu-CTC sequence (CCCTGGT), resembling the positional span of a canonical miRNA seed. These target binding sites match overlapping complementary sequences within the recurrent tRF-Glu-CTC sequence. However, binding site annotation was not provided by all studies, and some reported target genes without a specific binding region whereas others mentioned just a 3′UTR localization. This lack of evidence limited direct cross-study comparison of all interactions.

### 3.3. Database Cross-Referencing of tRF Sequences and Target Genes

Across the 18 studies, eight literature-reported tRF sequences showed full-length matches to tatDB entries, while 14 contained the exact recurrent 18-nt core sequence. Corresponding target entries were identified in tatDB for 14 studies, whereas tRFTar contained corresponding entries for five studies. The remaining comparisons involved partial or sequence-only overlaps, and three studies showed no corresponding match in either database under the stated criteria (Table 2). Comparison between the literature reports and database matches revealed a clear difference in the extent of sequence overlap. The literature-reported tRF-Glu-CTC isoforms show similarity with each other for up to 23 nucleotides. Matching tatDB entries contained an 18–23 nt 5′ core region, while the longer 3′ extension reported in the disease-specific studies was typically absent. Among the 18 studies, one exception showed a shorter 9 nt exact overlap. This exception corresponded to the target TYRO3 (Case 16), where the literature-reported sequence contained an additional 3′ extension beyond the region shared with the tatDB tRF. Thus, tatDB primarily supported recurrent overlapping sequence regions identified in CLASH results rather than the longer extended isoforms reported in smaller-scale papers (Table 2).

tRFTar showed a more limited agreement of tRF–target pairs with the literature, compared to tatDB, with support observed for five literature targets, including SRF [18], MARCKS [22], PABPC1 [25], and sequence-related entries for AKT2 [34] and CDK1 [31].

### 3.4. Recurrent tRF Sequence Overlap Is Observed in Large-Scale Experiments

Comparing the literature sequences with tatDB entries, we observed both full-length matches and partial sequence overlaps (Table 2). Eight literature-reported tRF sequences were fully represented within tatDB entries, while other cases involved shorter overlapping isoforms that often retained the recurrent 18 nt core. For example, in SRF and ARRB2 (name chosen based on tatDB hit), the reported sequence extends beyond 30 nt. tatDB contains isoforms Glu-CTC-001-N-5i-1–33 and Glu-CTC-003-N-5p-1–19/22. These isoforms preserve TCCCTGGTGGTCTAGTGG, the core region, but differ in the lengths of the 3′ termini, with truncation or extensions relative to the literature sequences.

Similarly, the literature sequence for VASH1 shows a substantial 3′ extension, while tatDB identifies an isoform Glu-TTC-003-N-5p-1–21, which retains the same 18 nt but includes only a short 3′ extension (CTA), lacking the remaining downstream 10 nt. For most of the targets, tatDB isoforms capture the 18–20 nt core sequence, while the remaining 3′ sequences show variability (Table 2).

For WDR1 [17], tatDB contained the core tRF sequence TCCCTGGTGGTCTAGTGG, associated with the target. In the case of FMOD, tatDB does not contain it as a direct target but contains an extended version of the tRF for targets MARCKS and GNA12, also reported as predicted candidate targets (but not validated) in the same study [22]. Also, tatDB contains the recurrent core sequence TCCCTGGTGGTCTAGTGG for predicted targets POLR2C [23] and AKT1 [24]. In contrast, APOER2 [20] and TAS1R3 [21] were reported as experimentally validated targets in their respective papers, but these same target genes were not found as direct tRF-Glu-CTC targets in either database.

### 3.5. Additional Evidence from Large-Scale Studies for Targets PABPC1 and SRF

**Figure 3:**
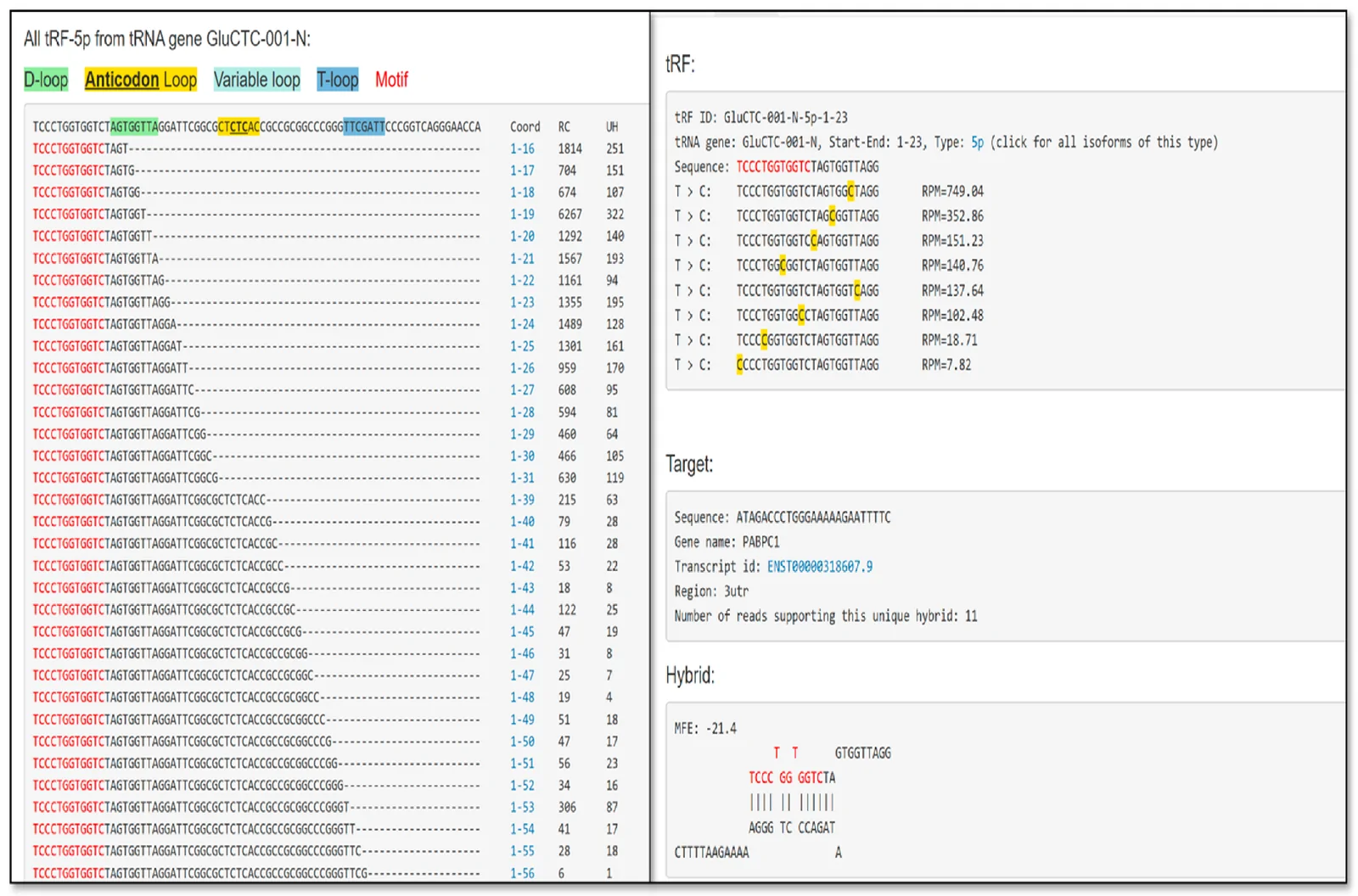
A snapshot of a results page from tatDB indicating binding between the tRF and the target PABPC1, suggested by CLASH. The (**left**) panel shows the 5′ tRF isoform GluCTC-001-N-5p-1–23 derived from the GluCTC-001 tRNA gene. Each tRF isoform is shown with its positional coordinates (Coord), read counts (RCs), and unique tRF–target hybrids (UHs). The selected 1–23 isoform was present in CLASH UH = 195 supported by RC = 1355. The (**right**) panel shows the tatDB details for one of the UHs containing the PABPC1-associated isoform, with the predicted target recognition sequence marked in red including target information, RNA-RNA hybrid structure, and minimum free energy (MFE = −21.4 kcal/mol). The illustrated PABPC1 hybrid was supported by 11 reads. Yellow marks indicate T > C conversion sites from PAR-CLIP data with their RPM values, discussed in text.

**Figure 4:**
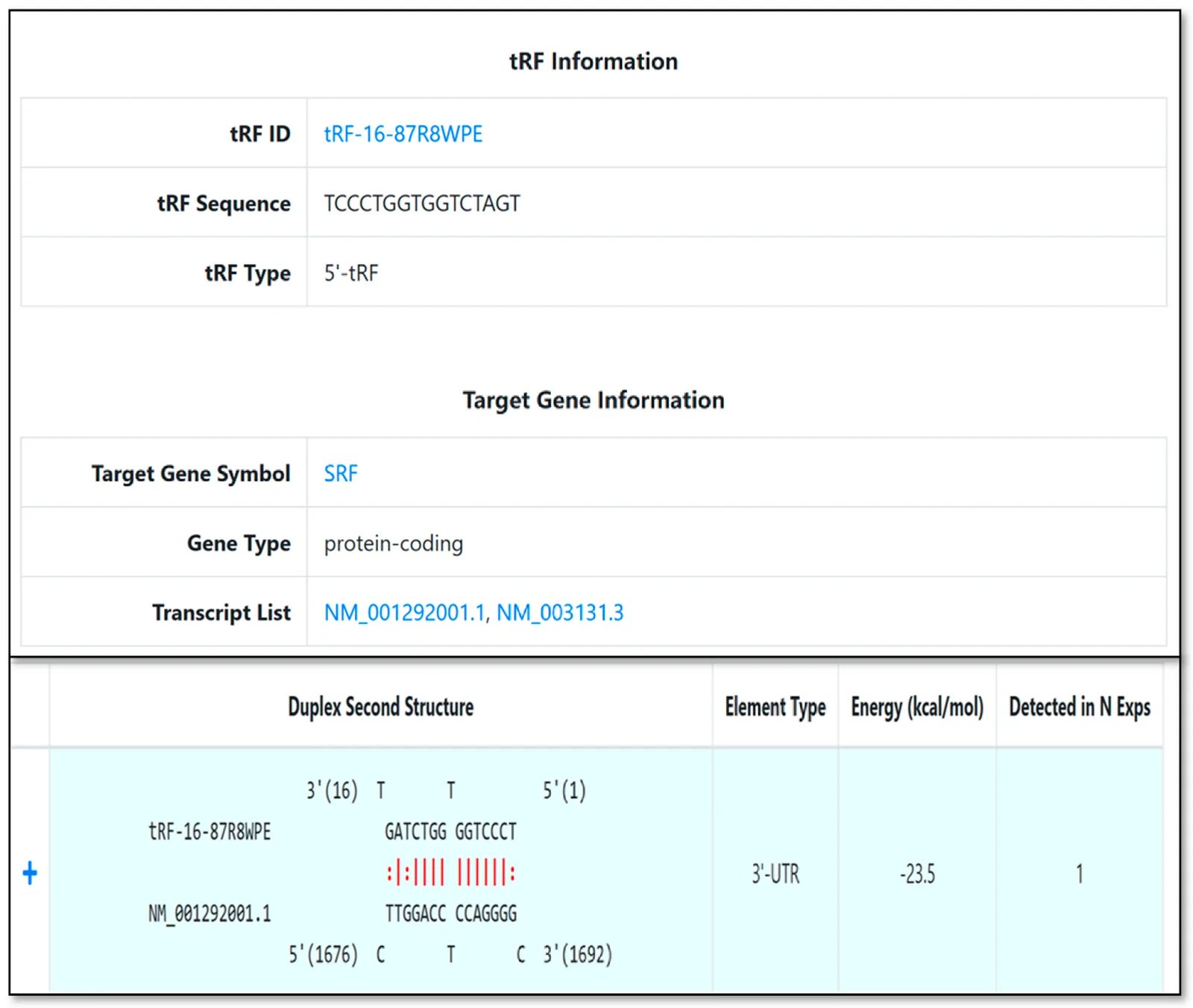
A snapshot of a results page from tRFTar indicating predicted binding between tRF-Glu-CTC and its target gene (SRF), along with the corresponding 3′UTR interaction and binding energy. The (**upper**) panel provides tRF information, including the tRF ID, sequence and tRF type. The selected tRF, tRF-16-87R8WPE, has the sequence TCCCTGGTGGTCTAGT and is annotated as a 5′ tRF. The (**middle**) panel shows target gene information for SRF, including protein-coding gene type and associated transcript IDs NM_001292001.1 and NM_003131.3. The (**lower**) panel shows the predicted RNA-RNA duplex between the tRF and the SRF 3′UTR, with the interaction located in the 3′UTR region. The predicted binding energy was −23.5 kcal/mol, providing a modeled estimate of duplex stability.

For several gene targets mentioned in the literature, the original studies did not clearly report the specific binding region complementary to the tRF seed sequence. Database support was observed for several literature-reported targets, including representative examples such as PABPC1 and SRF (Figure 3 and Figure 4).

Analysis of tRF-5p isoforms derived from the Glu-CTC-001 tRNA gene revealed extensive isoform heterogeneity, with multiple variants differing primarily in their 3′ terminal lengths while preserving the 5′ core sequence. Notably, the most abundant isoform (Glu-CTC-001-N-5p-1–23) showed high supporting read counts (RCs) of 1355 and multiple unique hybrids (UHs) of 195, indicating strong CLASH evidence of this tRF pairing with multiple targets. The modeled tRF–target duplex had a minimum free energy (MFE) of −21.4 kcal/mol, providing an estimate of predicted duplex stability. For PABPC1, tatDB provided a representative example of database-supported interaction involving a Glu-CTC-derived 5′ tRF isoform. The specific PABPC1-associated hybrid was supported by 11 reads and showed a predicted MFE of −21.4 kcal/mol.

The T > C conversion sites (yellow highlights in Figure 3) from PAR-CLIP data were also examined in relation to the likely target recognition region shown in tatDB (red letters in Figure 3). Among the eight T > C conversion sites for this isoform, four positions outside this target recognition region and two on the very edges had the overwhelming read support (total RPM > 1634, 98.4% of all RPM), compared to the two inside the region (seen as bulges). The distribution of conversion sites was examined descriptively in relation to the predicted target recognition region and was not considered functional confirmation of this region. Fewer conversions within the region may be compatible with AGO-associated contact sites involved in binding (Figure 3).

Similarly, tRFTar also supported a predicted interaction of the target gene SRF with this tRF. The tRFTar output showed tRF-16-87R8WPE, with the sequence TCCCTGGTGGTCTAGT, linked to SRF transcripts NM_001292001.1 and NM_003131.3. The predicted duplex was in 3′UTR and showed an MFE of −23.5 kcal/mol (Figure 4).

### 3.6. Target Homolog Analysis Suggests Candidate Target Site Similarity

**Figure 5:**
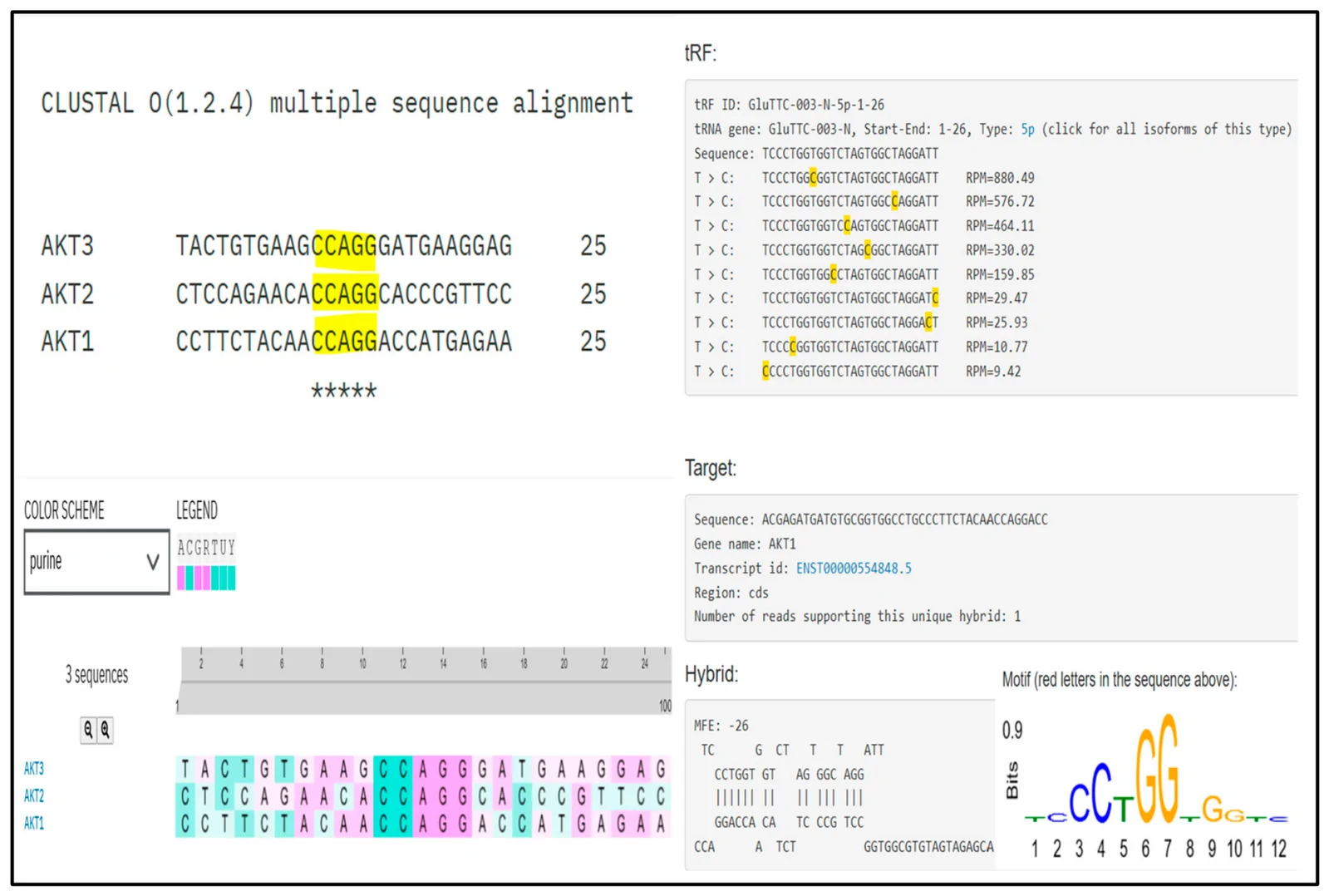
Analysis of AKT family genes to explore the targeting behavior of tRF-Glu-CTC. The (**left**) panel shows AKT1/2/3 3′UTR regions with the CCAGG-centered region highlighted. The (**right**) panel shows tatDB hybrid output, including tRF annotation, target sequence, transcript sequence, transcript region, hybrid structure, MFE value and sequence logo. Colors in the sequence alignment follow the Clustal Omega purine-based nucleotide color scheme; yellow highlighting indicates the CCAGG-centered region examined in this analysis.

We noted two homologs, AKT1 and AKT2, among the targets in Table 2 (both predicted) and considered (1) the possibility of erroneous gene identification in prediction or (2) a potential similarity in their target regions. Adding the third family member, AKT3, and using multiple alignment of 3′UTRs, we found that AKT1/2/3 all contain CCAGG (Figure 5), a reverse complement of the CCTGG subsequence in the tRF-Glu-CTC sequence. In tatDB, CCAGG-centered hybrid support was found for AKT1, but not for AKT2 or AKT3. Sequence and target site similarity may suggest a plausible regulatory relationship, but they do not provide direct evidence of interaction, so this example, while intriguing, should be considered just as a hypothesis-generating illustration. Also, the sequence logo visualization descriptively showed positional similarity across the aligned regions, reaching up to ~0.9 bits, with enrichment of cytosine and guanine residues within the core region (Figure 5). In contrast, members of other families (NSUN and SOD) did not show positionally recurrent sites.

## 4. Discussion

The recurrence of a highly similar tRF-Glu-CTC sequence reported in diverse disease contexts suggests that this tRF may participate in various regulatory processes and is repeatedly detected in different pathological conditions. Finding the same sequence core in studies of different tissues and species [10] is consistent with the expected similarity of potentially functional binding sites. Taken together, the 18 reports on tRF-Glu-CTC imply that it may be involved in post-transcriptional regulation of multiple target genes in processes such as cellular stress, injury or disease. As another indication of binding (observed in large-scale experiments), tatDB contains overlapping tRF-Glu-CTC subsequences detected in CLASH chimeras with the same targets as reported in disease-related papers. Eighteen studies, with only 14 indicating the binding region, are not sufficient for statistical inferences (but sequence identity extending to 18 nt is clear). We note that multiple genomic loci encode tRNA-Glu-CTC and could contribute to the observed isoform diversity. However, the shared short sequence among these isoforms alone is insufficient to establish an exact targeting rule, and functional relevance requires direct experimental validation.

tRFTar contains a small subset of literature targets of the Glu-CTC core, including SRF, MARCKS, PABPC1 and sequence matches for AKT2 and CDK1, whereas tatDB has a larger overlap with reported target interactions. This difference likely reflects variation in underlying experimental evidence and its computational processing, source datasets, exact tRF isoform annotation, and transcript coverage rather than simple presence or absence of a biologically relevant interaction. A key earlier observation that tRF-Glu-CTC represents a stable fragment is an important factor that likely contributes to its frequent detection in different conditions and cell types. The 5′ halves from glutamic acid tRNAs have been shown to exhibit enhanced resistance to nuclease degradation through dimerization contributing to their increased stability in biological systems [9]. This enhanced stability may support the fragment’s persistence, as it was detected in extracellular vesicles and other biofluids, potentially strengthening its detectability in disease studies [8], although this property needs more reliable evidence.

No common targets occurred in the literature reports; thus, strictly speaking, one should consider the possibility that this stability (and not biological function) may lead to an excess of tRF-Glu-CTC observed in the reported studies. However, the fact that tatDB lists >4000 of its targets detected in CLASH could provide a probabilistic explanation of why 18 studies, focusing on a few targets each, did not find common ones. And the majority of the targets were also found in CLASH, a very different experimental approach, complementary to these 18 papers.

Further, these fragments are reported in various species, including humans, mice, and rats [21,35]. The observed recurrence is consistent with the possibility that certain tRF-Glu-CTC isoforms represent reproducibly generated and persistently detectable fragments rather than isolated disease-specific degradation products. Glu-CTC-derived tRFs were among those with the highest numbers of CLASH targets, supporting reads, and unique tRF–target hybrids in our earlier motif-based analysis [16]. This high target representation, together with the reported structural stability of Glu-derived tRFs, may help explain the broad disease-associated target diversity observed despite no overlap among exact target genes. However, it remains unclear whether these numerous genes reflect the true biological target range or partly arise from CLASH and database-related artifacts [16].

The homolog-based comparison of the AKT family further highlights the importance of cautious interpretation. All three AKT family members have related CCAGG-containing site cores in 3′UTR regions, which are reverse complements of CCTGG present within the tRF-Glu-CTC sequence. Only AKT1 is present as a target in tatDB, while both AKT1 and AKT2 are predicted as smaller-scale studies. This highlights two points: (1) the absence of a potential target from a database does not necessarily exclude the presence of a related candidate target site and (2) sequence similarity and candidate target site similarity may suggest a possible regulatory relationship, yet they do not provide direct evidence of interaction. In contrast, analysis of other related gene families, such as NSUN2/NSUN4 and SOD2/SOD3, did not reveal comparably clear (or positionally common) target site patterns, as protein sequence homology generally does not correspond to similar tRF-Glu-CTC binding sites within UTRs.

Several disease studies included in the present analysis reported tRF-Glu-CTC or related tRNA-Glu-derived fragments in extracellular vesicles or exosome-associated contexts, including studies of pancreatic cancer [17], Huntington’s disease [26], preeclampsia [27], azoospermia [31] and intracranial aneurysm [32]. Taken together, these observations suggest that dimer-mediated stability may support persistence of tRNA-Glu-derived tRFs in extracellular environments. This may improve their detectability in EV-associated RNA fractions, as a stable fragment is more likely to be detected in biofluids than a rapidly degraded one [36].

Representative studies with direct functional validation further illustrate the range of evidence for individual tRF-Glu-CTC target interactions. For example, tRF-GluCTC-0005 was shown to bind the 3′UTR of WDR1 mRNA and increase its stability; this interaction was supported by RNA pulldown and luciferase reporter assays. In vivo experiments linked this interaction to hepatic stellate cell activation and pancreatic cancer liver metastasis [17]. Similarly, tRF-Glu-CTC suppressed VASH1 expression in endothelial cells, and wild-type/mutant luciferase assays identified functional interaction sites within the VASH1 3′UTR. Functional experiments further showed effects on endothelial migration, tube formation and choroidal neovascularization, although the VASH1 expression effect observed in vitro was not reproduced in vivo [19].

Recent studies further support the recurrence of Glu-CTC-derived tRFs in stress-associated diseases. 5′ tRF-Glu-CTC has been reported as elevated before seizures in plasma samples of temporal lobe epilepsy [37], in pancreatic β cells/islet resident macrophages under metabolic stress in type 2 diabetes [38] and in seminal plasma of oligozoospermic men [39]. Together, these observations illustrate the recurrent detection of tRF-Glu-CTC in various diseases, although its disease specificity and diagnostic performance require further validation. The broad disease distribution of tRF-Glu-CTC-associated targets also illustrates the diversity of biological processes represented in the compiled studies [10]. The targets represented in the present dataset include genes linked to proliferation, vascular remodeling, inflammation, oxidative stress, neurogenesis, viral response, and tissue remodeling. While these functions are diverse, they are frequently interconnected within disease-associated stress responses.

Several limitations should also be considered. First, our data collection was based on a curated set of published studies and therefore depends on completeness and reporting quality of the available literature, including the strength of target validation methods. The target validation strength varied substantially among studies. Evidence ranged from luciferase reporter assays indicating interactions to more sophisticated functional assays to primarily sRNA sequencing with rather simplistic bioinformatic follow-up without real validation. Also, the exact isoform boundaries and sequence annotations were not always reported consistently, complicating direct sequence comparisons. None of the studies cited here included motif enrichment, background frequency, positional enrichment, or matched non-target analysis. In the absence of direct functional validation for many of the reported interactions, the regulatory significance of the reported tRF-Glu-CTC studies remains speculative and will require further mechanistic investigation. Database searches may not match experimentally reported targets because of platform-specific curation, evidence thresholds or transcript-level differences. Literature-based analysis may also be affected by publication or reporting bias, while CLASH-derived or predicted findings may vary with experimental and database context. Our illustration of homolog-based target site comparisons, although useful for hypothesis generation, cannot establish functional tRF-mRNA interaction without direct experimental validation. Finally, the present study did not specifically assess naturally occurring sequence polymorphisms in tRF-Glu-CTC or its target binding regions. Although several studies used experimentally mutated target site constructs to test sequence-dependent interactions, these mutations are distinct from natural variants. Therefore, the effect of sequence polymorphisms on tRF–target complementarity remains to be determined and warrants further investigation.

## 5. Conclusions

tRF-Glu-CTC emerges as a recurrent candidate regulatory fragment that may be targeting mRNA sites with similar sequences in different cellular environments and diverse disease contexts. The reported interactions showed heterogeneous levels of evidence, ranging from computational prediction to direct site-level validation. Future work should focus on direct experimental validation of isoform-specific, disease-relevant tRF–target interactions and their cellular consequences.

## Figures and Tables

**Table 1:** tRF-Glu-CTC-reported targets in the literature, their validation methods with evidence tiers, and disease/condition. Evidence tiers, introduced in our earlier review [7], indicate the level of support for each reported target interaction: T1, computational prediction or association only; T2, functional target-level support; T3, direct site-level functional validation.

Case	tRF Name	Reference and Validation Method	Reported Target (s) and Evidence Level	Disease/Condition
1	tRF-GluCTC-0005	[17] RT-qPCR, RNA pulldown assay, dual-luciferase reporter assay, Western blot, immunofluorescence, cell migration and collagen contraction assays, mouse models, IHC, ELISA and flow cytometry	WDR1 (in vitro and in vivo); T3	Pancreatic cancer
2	tRF-Glu-CTC-014	[18] Atherosclerosis mouse model, small RNA sequencing, qPCR, Western blot, ELISA	ARRB (name provided in paper), SRF, (bioinformatic prediction; qPCR expression support); T1	Atherosclerosis
3	tRF-Glu-CTC	[19] RT-qPCR, immunofluorescence, dual-luciferase reporter assay, fundus fluorescein angiography, ex vivo choroidal sprouting assay, endothelial cell proliferation, migration and tube formation assays, transcriptome sequencing	VASH1 (in vitro); T3	Age-related macular degeneration
4	tRF5-GluCTC	[20] Northern blot, qRT-PCR, Western blot, RNA pulldown/HITS-Abt (High-Throughput Sequencing of RNAs Associated with Biotinylated tRF), dual-luciferase reporter assay, RNA interference, co-immunoprecipitation, virus titration assay	APOER2 (in vitro); T3	Respiratory syncytial virus infection
5	tRF-Glu-CTC-013	[21] tRF/tiRNA sequencing, RT-qPCR, dual-luciferase reporter assay, Western blot, autophagy and autophagic flux assays, histological staining/cardiomyocyte area analysis	TAS1R3 (in vitro); T3	Cardiac hypertrophy
6	tRF-1:29-Glu-CTC-1	[22] Small RNA sequencing, qRT-PCR, ELISA, dual-luciferase reporter assay, Western blot, EdU cell proliferation assay, wound healing, Transwell migration assay, rat carotid balloon injury model, histological and immunohistochemical analysis	FMOD validated (in vitro and in vivo); T3 MARCKS, GLP1R, GNA12 mentioned as targets (bioinformatic prediction); T1	Vascular remodeling/neointimal hyperplasia
7	tDR-001276	[23] Small RNA sequencing, qRT-PCR	POLR2C, MAP3K7 (bioinformatic prediction); T1	Fibrous dysplasia
8	tRF-Glu-CTC-003	[24] Small RNA sequencing, qRT-PCR, cell supernatant analysis, plasma exosome analysis, paired tissue analysis, agarose gel electrophoresis	AKT1 (bioinformatic prediction); T1	Early-stage breast cancer
9	tRF5-GluCTC	[25] tRF pulldown, mass spectrometry/proteomics, Western blot, immunoprecipitation, qRT-PCR, siRNA knockdown, virus titration assay and immunostaining, cytokine analysis	PABPC1 (in vitro; protein-binding interaction); T2	Respiratory syncytial virus infection
10	tRF-Glu-CTC	[26] Plasma EV isolation, flow cytometry, nanoparticle tracking analysis, Western blotting, cryogenic transmission electron microscopy, small RNA sequencing, qRT-PCR validation—to mostly validate tRF as a biomarker, not to find its targets	Among the many genes predicted computationally: MAGI2, PDE4D (bioinformatic prediction); T1	Huntington’s disease
11	5′-tRF-Glu-CTC	[27] Placental perfusion, EV isolation, nanoparticle tracking analysis, Western blotting, transmission electron microscopy, small RNA sequencing, RT-qPCR, tRF transfection in monocytes/macrophages and endothelial cells, macrophage-conditioned-medium assay	Pathway genes: ICAM1, VCAM1, NOS3 (in vitro; downstream expression effects); T2	Preeclampsia
12	tRF-Glu-CTC	[28] CGR8 embryonic stem cell culture and differentiation to NSC (neural stem cells), qRT-PCR, immunofluorescence, H_2_O_2-_induced NSC model, detection of H_2_O_2_–zebrafish embryos, HyPer fluorescence imaging, RNA probe synthesis, whole-mount in situ hybridization	MAP2, GFAP (in vitro; downstream expression markers); T2	Neurogenesis
13	AS-tDR-013428	[29] Brain-aging mouse model, behavioral assessment, small RNA sequencing, qPCR	RPSA (bioinformatic prediction); T1	Neurodegenerative disorders such as Alzheimer’s disease and Parkinson’s disease
14	tRF5-Glu	[30] qRT-PCR, Northern and Western blot, transfection with siRNA, dual-luciferase reporter assay, miR-Catch and BCAR3 mRNA pulldown, Sulforhodamine B proliferation assay	BCAR3 (in vitro); T3	Ovarian cancer
15	tRF-Glu-CTC-005	[31] Plasma exosome isolation, transmission electron microscopy, particle size/concentration analysis, exosomal RNA extraction, agarose gel electrophoresis, small RNA sequencing, RT-qPCR	RBX1, CDK1 (bioinformatic prediction); T1	Azoospermia
16	tRF-13-33-GluCTC	[32] Plasma exosome isolation, transmission electron microscopy, nanoparticle tracking analysis, sRNA sequencing, RT-qPCR, fluorescence in situ hybridization, Western blot, flow cytometry, reactive oxygen species staining, dual-luciferase reporter, TUNEL assay, immunofluorescence	TYRO3 (in vitro and in vivo); T3	Intracranial aneurysm
17	tRF-33-87R8WP9N1EWJDW	[33] Serum small RNA isolation, miR-Q RT-qPCR, RT-qPCR and peripheral blood mononuclear cell isolation, IL-1Ra-knockout mouse serum analysis, plasma EV isolation by size exclusion chromatography, nanoparticle tracking analysis	NSUN2, SOD2 (clinical/expression analysis; not direct target validation); T1	Psoriatic arthritis
18	tRF-1:24-Glu-CTC-1-M2 (tRF-1:2)	[34] Podocyte cell culture, high glucose injury model, RT-qPCR, high-throughput sequencing, Western blotting	AKT2 (bioinformatic prediction); T1	High glucose-induced podocyte injury in kidneys

**Table 2:** Comparison of tRF-Glu-CTC sequences with reported database-matched regions. The sequences are mentioned in the same order as the corresponding references listed in Table 1. Underlined nucleotides indicate the exact nucleotide match between the literature-reported tRF sequence and the corresponding sequence in databases (tatDB and tRFTar), with a separate column of potential Binding Region (if provided, otherwise “Not given”) in the 18 studies of Table 1. Truncated or extended isoforms were considered when they shared an exact overlapping sequence. “Not found” indicates that no corresponding sequence or target gene name was identified under the stated database version and query criteria. All reported mRNA binding sites were located in 3′UTR. No site locations were reported for PABPC1, MAGI2, PDE4D, VCAM1, MAP2, GFAP, RPSA, BCAR3, RBX1, CDK1, TYRO3, NSUN2, SOD2, and AKT2.

Case	Sequence in Paper (5′–3′)	Binding Region	tatDB tRF for Matching Target(s)	tRFTar tRF for Matching Target(s)
1	TCCCTGGTGGTCTAGTGGTTAGGAT	ATCAGGGA	TCCCTGGTGGTCTAGTGGTTAGGATTCGGCGCT	Not found
2	TCCCTGGTGGTCTAGTGGTTAGGATTCGGCG	ACCAGGG	TCCCTGGTGGTCTAGTGGTTAGGATTCGGCGCT for SRF and ARRB2	TCCCTGGTGGTCTAGT for SRF
3	TCCCTGGTGGTCTAGTGGTTAGGATTCGGCG	CCAGGG	TCCCTGGTGGTCTAGTGGCTA	Not found
4	TCCCTGGTGGTCTAGTGGTTAGGATTCGGCG	ACCAGGGA	Not found	Not found
5	TCCCTGGTGGTCTAGTGGTTAGGATTCGGC	CACCAGGGA	Not found	Not found
6	TCCCTGGTGGTCTAGTGGTTAGGATTCGG	ACCAGGG	TCCCTGGTGGTCTAGTGGTTAGGATTCGGCGCT for MARCKS and GNA12	TCCCTGGTGGTCTAGTGGTTAG for MARCKS
7	TCCCTGGTGGTCTAGTGGCTAGGATTCGGCGCTT	ACCAGGG	TCCCTGGTGGTCTAGTGGTTAGGA for POLR2C and TCCCTGGTGGTCTAGTGGTTAGGATTCGGCGCT for MAP3K7	Not found
8	TCCCTGGTGGTCTAGTGG	ACCAGGG	TCCCTGGTGGTCTAGTGGCTA for AKT1	Not found
9	TCCCTGGTGGTCTAGTGGTTAGGATTCGG	Not Given	TCCCTGGTGGTCTAGTGGTTAGGATTCGGCGCT for PABPC1	TCCCTGGTGGTCTAGTGGTTAGG for PABPC1
10	TCCCTGGTGGTCTAGTGGTTAGGATTCGGCG	Not Given	TCCCTGGTGGTCTAGTGGCTA for both MAGI2, PDE4D	Not found
11	TCCCTGGTGGTCTAGTGGTTAGGATTCGGCGCT	Not Given	TCCCTGGTGGTCTAGTGGCTAGGA for VCAM1	Not found
12	TCCCTGGTGGTCTAGTGGTTAGGATTCG	Not Given	TCCCTGGTGGTCTAGTGGTTAGGATTCGGCGCT for MAP2, TCCCTGGTGGTCTAGTGGCTAG for GFAP	Not found
13	TCCCTGGTGGTCTAGTGGTTAGGATA	Not Given	TCCCTGGTGGTCTAGTGGTTAGGATTCGGCGCT for RPSA	Not found
14	CCCTGTGGTCTAGTGGTTAGGATTCGGCGCTCTC	Not Given	Not found	Not found
15	Not given	Not Given	TCCCTGGTGGTCTAGTGGTTAGGATCGGCGCTCTCA for CDK1	CDK1 target present but not based on the target sequence
16	TAGTGGTTAGGATTCGGCGCT	Not Given	TCCCTGGTGGTCTAGTGGTTA for TYRO3	Not found
17	TCCCTGGTGGTCTAGTGGTTAGGATTCGGCGCT	Not Given	NSUN4 and SOD3 mentioned, NSUN2 and SOD2 validated. TCCCTGGTGGTCTAGTGGTTAGGATTCGGCGCT for NSUN4	Not found
18	TCCCTGGTGGTCTAGTGGTTAGGA	Not Given	TCCCTGGTGGTCTAGTGGTTAGGATTCGGCGCT for AKT2	AKT2 target present but not the same sequence

## Data Availability

Data included in the article.

## Abbreviations

The following abbreviations are used in this manuscript:

AGO	Argonaute
BLAST	Basic Local Alignment Search Tool
CDS	Coding sequence
CLASH	Crosslinking, Ligation, and Sequencing of Hybrids
ELISA	Enzyme-Linked Immunosorbent Assay
EV	Extracellular vesicle
IHC	Immunohistochemistry
kcal	Kilocalorie
MFE	Minimum free energy
PAR-CLIP	Photoactivatable Ribonucleoside-Enhanced Crosslinking and Immunoprecipitation
qPCR	Quantitative Polymerase Chain Reaction
RC	Read count
RISC	RNA-induced silencing complex
RPM	Reads per million
RT-qPCR	Reverse Transcription Quantitative Polymerase Chain Reaction
sRNA	small RNA
tRF	tRNA-derived fragment
TUNEL	Terminal deoxynucleotidyl transferase dUTP nick end labeling
UH	Unique hybrid
UTR	Untranslated region

## Appendix A

**Table A1:** Reported target genes and their biological functions (obtained from https://www.genecards.org; last accessed 21 August 2026). # indicates the case number corresponding to Table 1.

#	Reported Target Genes	Target Gene Function
1	WDR1	Actin filament disassembly; involved in cytokinesis and cell migration
2	SRF, ARRB2	SRF: Transcription factor regulating genes involved in development and cell migration; ARRB2: regulates G-protein-coupled receptor signaling and receptor desensitization/resensitization
3	VASH1	Angiogenesis inhibitor; inhibits endothelial cell migration and proliferation
4	APOER2	Receptor involved in DAB1 phosphorylation and neuronal microtubule function
5	TAS1R3	Glucose and amino acid sensing receptors that regulate mTORC1 signaling, autophagy, inflammation and cardiac hypertrophy
6	FMOD, MARCKS, GLP1R, GNA12	FMOD: Regulates collagen fibrillogenesis and the rate of collagen fibril formation; MARCKS: structural modulation of actin cytoskeleton involved in motility, chemotaxis and cell adhesion; GLP1R: G-protein that regulates insulin secretion; GNA12: G-protein in signal transduction and RhoA signaling
7	POLR2C, MAP3K7	POLR2C: Core component of RNA polymerase II involved in transcription of mRNA and noncoding RNAs; MAP3K7: serine threonine kinase and essential component of MAP kinase signaling
8	AKT1	Serine/threonine kinase regulating metabolism, cell survival, growth and proliferation
9	PABPC1	Binds mRNA poly(A) tails and regulates mRNA metabolism and stability
10	MAGI2, PDE4D	MAGI2: Scaffold protein assembling receptors and cell adhesion proteins at synaptic junctions; PDE4D: hydrolyzes the second messenger cAMP
11	ICAM1, VCAM1, NOS3	ICAM1: Cell adhesion molecule involved in leukocyte cell–cell adhesion and immune responses; VCAM1: endothelial cell adhesion protein involved in immune surveillance and inflammation; NOS3: produces nitric oxide involved in vascular smooth-muscle relaxation
12	MAP2, GFAP	MAP2: Microtubule-associated protein involved in stabilization of microtubules; GFAP: class III intermediate filament protein and astrocyte marker CNS development
13	RPSA	Component of the 40S ribosomal subunit; also functions as a laminin receptor
14	BCAR3	Adapter protein promoting cell proliferation and migration downstream of growth factor receptors
15	RBX1, CDK1	RBX1: Core component of RING-type E3 ubiquitin ligase complexes mediating protein ubiquitination; CDK1: cyclin-dependent kinase and key regulator of cell cycle progression
16	TYRO3	Receptor tyrosine kinase regulating cell survival, migration and differentiation
17	NSUN2, SOD2	NSUN2: RNA cytosine C5-methyltransferase involved in RNA methylation and stability; SOD2: mitochondrial enzyme that removes toxic superoxide radicals
18	AKT2	Serine/threonine kinase regulating metabolism, proliferation, cell survival, growth and angiogenesis
